# Simulating eutrophication in a metacommunity landscape: an aquatic model ecosystem

**DOI:** 10.1007/s00442-018-4319-8

**Published:** 2018-12-06

**Authors:** Josie Antonucci Di Carvalho, Stephen A. Wickham

**Affiliations:** Department of Ecology and Evolution, University of Salzburg, Hellbrunnerstrasse 34, 5020 Salzburg, Austria

**Keywords:** Interconnected patches, Diversity, Fragmentation, Nutrient addition, Microcosm experiment

## Abstract

Aquatic habitats are often characterized by both high diversity and the threat of multiple anthropogenic stressors. Our research deals with temporal and spatial aspects of two of the main threats for biodiversity, namely eutrophication and fragmentation. It is known that pulsed nutrient addition creates temporal differences in environmental conditions, promoting higher diversity by preventing the best competitor from dominating. Furthermore, a metacommunity landscape with intermediate connectivity increases autotrophs’ diversity and stability. However, it is yet unclear if these two factors are additive in increasing diversity and if the effects extend to the consumer level. With the goal of understanding how eutrophication impacts biodiversity in a metacommunity landscape, we hypothesized that pulsed nutrient addition will increase diversity among both autotrophs and heterotrophs, and this effect will be even greater in a metacommunity landscape. We simulated eutrophication and fragmentation in a microcosm experiment using phytoplankton as primary producers and microzooplankton as grazers. Four treatment combinations were tested including two different landscapes (metacommunity and isolated community) and two forms of nutrient supply (pulsed and continuous): metacommunity/continuous nutrient addition (MC); metacommunity/pulsed nutrient addition (MP); isolated community/continuous nutrient addition (IC); isolated community/pulsed nutrient addition (IP). As expected, pulsed nutrient addition had a persistent positive effect on phytoplankton diversity, with a weaker influence of landscape type. In contrast, the grazer community strongly benefited from a metacommunity landscape, with less significance of pulsed or continuous nutrient addition. Overall, the metacommunity landscape with pulsed nutrient supply supported higher diversity of primary producers and grazers.

**Electronic supplementary material:**

The online version of this article (10.1007/s00442-018-4319-8) contains supplementary material, which is available to authorized users.

## Introduction

Community ecology has long focused on processes that regulate patterns of species distribution and abundance. Not only have these processes taken on new significance in an era with significant biodiversity loss, but it has also become clear that maintaining biodiversity is essential for maintaining ecosystem functions (Tilman et al. 2001). Local and regional biodiversity measurements are known to be important tools to understand ecosystem dynamics for further application on environmental issues (Resetarits et al. 2018). The dispersal of organisms at the regional scale can counter local diversity loss, taking on an important role in ecosystem productivity and stability (Holyoak et al. 2005). Model aquatic systems have long been used to test metacommunity theory, but they are applicable to real aquatic environments, even those that have constantly changing connectivity between local patches, such as interconnected lagoons, estuarine lakes and salt ponds (Smeti et al. 2016).

It has been shown that higher diversity results in greater stability, higher productivity and better resource use efficiency by increasing either the likelihood that at least one high-productivity species is present, or the number of niches that can be occupied (Tilman and Downing 1994; Tilman et al. 2001; Cardinale 2011). When higher species richness leads to more efficient resource use and, therefore, higher community biomass, this can stabilize communities by reducing demographic stochasticity (de Mazancourt et al. 2013). Despite its many benefits for ecosystems, biodiversity is increasingly under threat. The present research deals with the temporal and spatial aspects of two of these threats: habitat fragmentation and eutrophication.

It was already clear to MacArthur and Wilson (1967) that habitat fragmentation can lead to local extinction, and if there is no connection between newly isolated patches, the local extinction can occur remarkably quickly (Gibson et al. 2013). Higher trophic levels are known to be more sensitive to landscape changes, mainly because of their larger body size and lower richness, which can result in the alteration of ecosystem functions driven by top-down extinction (Duffy 2003; Chisholm et al. 2011). However, the connectivity between fragments plays an important role in mitigating the biodiversity loss, allowing the dispersal of organisms among the patches (Staddon et al. 2010; Chisholm et al. 2011). If patches are connected to an appropriate degree, building a metacommunity, the regional (across patch) community can persist longer than a single large patch (Holyoak and Lawler 1996; Holyoak 2000a). Here, we tested this prediction, predicting longer survival of organisms in smaller interconnected patches (metacommunity) than in a larger isolated habitat.

There is a consensus that local biodiversity is generally highest at an intermediate productivity (Kassen et al. 2000; Hillebrand and Lehmpfuhl 2011). This implies that eutrophication (anthropogenically caused increases in nutrient addition) can have positive effects on biodiversity in systems that were originally low-productive, or negative effects if the water body already had medium to high nutrient loadings (Heino 2013). Habitats with originally low nutrient concentrations tend to be highly sensitive towards small changes in nutrient enrichment (Taylor et al. 2014), which can result in primary producer shifts affecting subsequently the composition of higher trophic levels (Cook et al. 2018). At a regional scale, however, aquatic biodiversity has been found to increase continuously with increasing productivity as more productive patches (ponds in this case) were more dissimilar to one another than at low productivity (Chase and Leibold 2002). It is plausible, but still untested, that a metacommunity of patches with varying productivity would result in higher diversity as different nutrient concentrations among patches would allow different species to survive.

Despite the existence of many studies on dispersal effects and nutrient loading (pulsed or continuous), these issues have been largely approached separately and commonly using only one trophic level. In this study, we addressed two less understood aspects: the combined effect of dispersal and nutrient addition in influencing biodiversity and productivity, and their effects on grazers and primary producers. Experimental work, using only phytoplankton assemblages, has shown that diversity is more influenced by species characteristics than by dispersal rate (connectivity) in a metacommunity structure (Smeti et al. 2016). Nutrient availability in aquatic systems influences diversity and biomass of primary producers and, moreover, it can act as a disturbance when it is loaded in a pulsed way, influencing the growth and losses of the populations (Roelke 2000; Roelke and Eldridge 2007). The pulsed inflows create environmental changes through the temporal fluctuations of resources, allowing different species to coexist according to their abilities to survive (Roelke and Spatharis 2015; Smeti et al. 2016; Papanikolopoulou et al. 2018). Pulsed nutrient addition has long been known to increase phytoplankton diversity by preventing competitive dominance under equilibrium conditions (Sommer 1985). Furthermore, the benefit of intermediate connectivity in a metacommunity landscape in increasing primary producers’ diversity is also known (Limberger and Wickham 2012a; Steiner et al. 2013). However, it is yet unclear if those two factors are additive in increasing diversity and if the grazers’ responses are related to their prey.

Here, we aimed to elucidate the interaction of nutrient addition and habitat fragmentation (landscape) in influencing biodiversity. To achieve this goal, we tested two different landscapes, isolated and interconnected communities, in a microcosm experiment. The landscape treatment was crossed with nutrients supplied either daily (continuously) or weekly (pulsed), resulting in four treatment combinations: metacommunity/continuous nutrient addition (MC); metacommunity/pulsed nutrient addition (MP); isolated community/continuous nutrient addition (IC); isolated community/pulsed nutrient addition (IP). The species used as model organisms consisted of three species of algae and cyanobacteria as primary producers, and six ciliates and rotifer species as grazers. Prior to the experiment, the grazers were established to be true competitors for the autotrophs used as their resource. The initial environmental conditions and species composition of all patches were identical. This research seeks to investigate the following hypotheses: By increasing the temporal variability in nutrient concentrations, pulsed nutrient addition will increase diversity not only among autotrophs (Sommer 1985) but also in their grazers (H_1_); additionally, these effects are expected to be even greater in a metacommunity landscape (in the MP treatment). The fragmented patches, interconnected by dispersal (metacommunity) and receiving weekly nutrient supply, would have yet higher diversity, as temporal niche differentiation (due to differences in nutrient concentrations) would allow more species to coexist (H_2_). However, we expected stronger effects in the grazer community, since only one species of phytoplankton was actively mobile. Therefore, landscape effects on autotrophs are expected to be primarily through the top-down grazing pressure (H_2_′); we further predicted that the metacommunity patches under pulsed supply would have higher beta-diversity (here measured as Bray–Curtis dissimilarities) than the patches under continuous nutrient addition, as the time interval between the pulses would accentuate stochasticity in nutrient availability, resulting in higher dissimilarity among patches (H_3_); finally, we predicted that higher community biomass and higher zooplankton:phytoplankton biomass ratio (resource use efficiency—RUE) would be promoted by metacommunities with pulsed nutrient addition (H_4_). More specifically, we expected highest diversity measures (local, regional and beta-diversity) as well as highest biomass and abundance in MP treatments, followed by MC treatments. Consecutively, IP and IC treatments are expected to have lower local diversity and standing stock measures (biomass and abundance) than the metacommunities, with even lower values in the latter.

## Materials and methods

### Species

The model organisms used in the experiment belong to two different trophic levels, primary producers (phytoplankton) and grazers (microzooplankton). Phytoplankton consisted of two species of algae and one species of cyanobacteria, respectively: *Desmodesmus abundans, Cryptomonas sp.* (strain SAG 26.80) and *Synechococcus sp*. The zooplankton community was composed of four ciliate species (*Coleps hirtus, Paramecium bursaria, Halteria sp., Stylonychia sp.*) and two rotifer species (*Lepadella sp.* and *Keratella cochlearis*). The aim of having three prey species was to provide a mixture of food quality for the grazer community.

The grazers were isolated from ponds located in Hans-Donnenberg-Park, Salzburg, Austria. Three months prior to the commencement of the experiment the species were isolated and maintained in 6-well plates with Volvic© water and algae for culturing, with transfers to new media roughly twice a week. These procedures continued until the desired abundance of each species was achieved. The autotrophs species had been originally obtained from the culture collection at Göttingen (SAG) and had been in culture in our laboratory for several years.

All species of autotrophs and heterotrophs were added to each bottle in equivalent biomass. The grazers’ and the primary producer’s biovolume was estimated from geometric formulas and converted to biomass using carbon conversion factors from the literature (Rocha and Duncan 1985; Stemberger and Gilbert 1987; Putt and Stoecker 1989).

### Experimental design

#### Landscape

The design of the experiment comprised two different landscapes, namely metacommunities and isolated communities. The former were represented by four 125 mL polycarbonate bottles, interconnected with silicon tubes (0.5 cm inner diameter and 40 cm length), allowing active dispersal of the zooplankton species. In this landscape, each bottle was connected with its other two neighboring bottles, in the form of a square. The isolated communities were represented by single 640 mL capacity polycarbonate bottles, filled to the same total volume as the 4 smaller bottles in the metacommunity landscape. The dispersal ability of the zooplankton species was verified in a preliminary experiment, in which two bottles, with equal volumes of 80 mL, were interconnected by silicone tubing of 40 cm length. One bottle contained 500 individuals of each zooplankton species + medium and the other bottle contained only medium. The initially empty bottle was sampled every 24 h, and migration of the slowest species could be confirmed after 72 h of experiment.

#### Nutrient addition

We added phosphorus and nitrogen at the Redfield (1958) ratio in the respective concentrations, per bottle: *P*: 0.016 µmol P L^−1^ day^−1;^*N*: 0.258 µmol N L^−1^ day^−1^. Continuous nutrient addition was performed by adding 1.53 mL of nutrients to each metacommunity bottle (10.71 mL week^−1^ in each bottle) using three multi-channel peristaltic pumps, active 30 min per day. In the isolated communities, 6.12 mL of nutrients (1.53 mL × 4) was delivered per day in each replicate bottle, during the same time period (30 min).

The pulsed nutrient addition was conducted weekly using a pipette. This interval corresponds to approximately 3–10 generations of the autotrophs and grazers and was chosen for being long enough to allow possible stochasticity to play a role, without inducing severe nutrient limitation. An equal volume of nutrients was added to all four patches at the same time. The volume added per week was the same total volume of nutrients added continuously. This means that per week each metacommunity bottle was supplied with 10.71 mL of nutrients, and each isolated community bottle received 42.84 mL of nutrients, either continuously or pulsed. The volume of nutrients added was such that all treatments received the same total nutrient addition (divided between four bottles in the metacommunity treatments, or all into the single bottle of the isolated treatment), with only the timing of the nutrient addition varying between the pulsed and continuous treatments.

We compared four different treatment combinations, hereafter: metacommunity supplied with continuous nutrient addition—MC; metacommunity with pulsed nutrient addition—MP; isolated community with continuous nutrient addition—IC; isolated community with pulsed nutrient addition—IP. The experiment setup comprised 30 bottles: 2 metacommunity treatments (with 4 bottles each) × 3 replicates + 2 local isolated communities (1 bottle each) × 3 replicates.

The initial volume of each metacommunity patch was 80 mL, with additional 10 mL in each connecting tube. The isolated communities had an initial volume of 360 mL, which corresponded to the volume of an entire metacommunity. We first filled the bottles with Volvic© mineral water, then we added the nutrients and, finally, the organisms. The experiment was conducted in a laboratory with a constant temperature of 20 °C on a 12:12 light:dark cycle with light intensity high enough to ensure algal growth (ca. 200 µE).

The experiment was carried out for 6 weeks and was sampled weekly. The sampling volume was 10 mL of each metacommunity bottle and 40 mL of the isolated community bottles. Since the sampling volume was roughly balanced by the total volume of nutrients added per week, it was not necessary to replace it. The tubes connecting the metacommunities were clamped before the sampling to avoid unintended active dispersal of the organisms. The samples were fixed with Lugol´s iodine solution. The volume of sample analyzed varied between 1 and 3 mL, depending on the abundance of species observed. The individuals were counted under an inverted microscope.

### Statistical analyses

All statistical analyses were performed with R (R version 3.4.1), using the vegan (Oksanen et al. 2017), dplyr (Wickham and Francois 2016), betapart (Baselga and Orme 2012), ecodist (Goslee and Urban 2007), MASS (Venables and Ripley 2013), nlme (Pinheiro 2018), multcomp (Hothorn et al. 2008) and lsmeans (Lenth 2016) packages.

#### Biodiversity estimation

Diversity was calculated as Shannon–Wiener index (here also called simply as diversity), richness and evenness. These measures were estimated separately for heterotrophs and autotrophs, at regional and local scales. Regional Shannon, richness and evenness were calculated only for the metacommunity landscape, in which the mean abundance of species was calculated among the four patches, and the diversity measure was estimated for the entire metacommunity. Local Shannon, richness and evenness were calculated for the two landscape types by calculating each diversity measure for each patch based on the local abundance of species. For metacommunities, the local diversity measure was then averaged among the four patches. The differentiation between regional and local scales was not possible for isolated communities since this landscape was formed with only one patch. Therefore, the results for metacommunities are depicted in two scales, whereas the results of isolated communities are shown only at the local scale.

#### Dissimilarity among metacommunity patches and the beta-diversity components

Beta-diversity was estimated for metacommunity landscape to investigate the dissimilarity among the four patches, under the two temporal nutrient additions. The components of beta-diversity, i.e., spatial turnover and nestedness, were also distinguished, based on Baselga and Orme (2012) and Baselga (2013). Disentangling these components is considered important for planning conservation strategies (Heino 2011). Nestedness can be defined as the process of addition/removal of species (Angeler 2013) and it is found when sites with smaller number of species are subsets of the richer sites. In contrast, turnover is characterized by the replacement of species that can occur as a result of environmental conditions (Wright and Reeves 1992; Ulrich and Gotelli 2007; Baselga 2010).

The dissimilarity in species composition of two or more sites can be defined using a qualitative (species presence-absence) or a quantitative (species abundance) criterion (Baselga 2013). In our experiment, we investigated the temporal changes among the patches based on the quantitative criterion, counting the abundance of zooplankton and phytoplankton for each week, with the aim of analyzing the species community as a whole. Then, the dissimilarities were measured as Bray–Curtis index using the *beta.multi.abund.R* function, which computes three multiple-sites dissimilarities accounting for: balanced variation in abundances or turnover; abundance gradients or nestedness; and beta-diversity or total dissimilarity (Baselga 2017). The dissimilarity values obtained from the Bray–Curtis metric vary from 0, indicating total similarity between two sites; to 1, which indicates that the sites are of maximum dissimilarity. Additionally, when there is no abundance gradient between sites (no nestedness), 0 indicates equal species abundance between sites and 1 indicates maximum dissimilarity of species and the abundances of species are balanced between the sites (maximum turnover); when there is no balanced variation in species abundance (no turnover), 0 also indicates no difference in species abundance between sites, and 1 indicates strong dissimilarity in species abundance but these differences are not balanced between sites—maximum nestedness (Baselga 2013, 2017).

#### Effects of “nutrient addition” and “landscape” on local, regional and beta-diversity

Two-way ANOVA with repeated measures was used to test the effects of nutrient addition and landscape on Shannon diversity, richness, evenness and biomass of heterotrophs and autotrophs. Differences between regional and local scales were also computed when applicable. Time was used as the within-subject variable; and nutrient addition and landscape were used as the two between-subject variables. Furthermore, ANOVA with repeated measures was also used to test the effects of nutrient addition on beta-diversity in metacommunity landscapes. In this case, the data of zooplankton and phytoplankton were computed together and only the nutrient addition was used as a between-subject variable. *p* values were evaluated considering the significance threshold at 0.05. When interactions between treatment factors were significant, Tukey’s post hoc tests were applied to better investigate the relations encountered.

#### Biomass ratio between zooplankton and phytoplankton

Resource use efficiency (RUE) was calculated as the biomass ratio between the primary producers and the grazers. The biomass ratio was estimated for the four treatment combinations, over the 6 weeks of experiment. Local and regional RUE were calculated for metacommunities and local RUE was calculated for isolated communities.

## Results

### Biodiversity

**Fig. 1:**
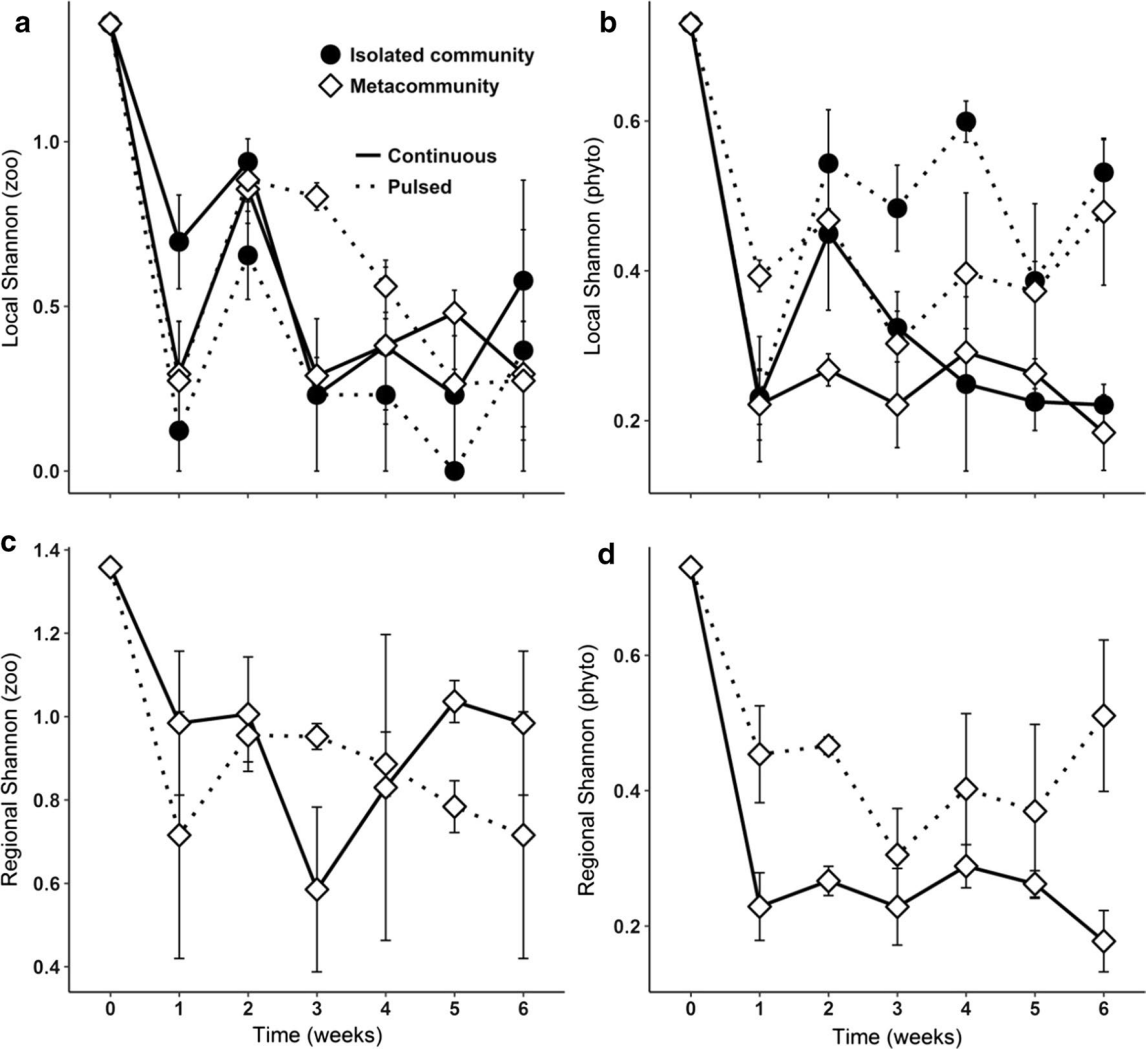
Shannon diversity of zooplankton and phytoplankton. Local Shannon diversity of zooplankton (**a**) and phytoplankton (**b**) were estimated for metacommunities and isolated communities. Regional Shannon diversity of zooplankton (**c**) and phytoplankton (**d**) were estimated only for metacommunities. Metacommunities are represented by open diamonds and isolated communities are represented by filled circles. Continuous nutrient addition is represented by solid lines and pulsed nutrient addition is represented by dotted lines. Values are mean ± SE, *n* = 3

Nutrient addition significantly affected producers’ Shannon diversity (Tables 1 and 2), while it had a weaker effect on grazers´ diversity, and was dependent on the landscape design (Table 1). Agreeing partly with our first hypothesis (H_1_), pulsed nutrient addition resulted in higher phytoplankton diversity compared to continuous nutrient addition, independently of the landscape type and persistently over time (Fig. 1b, d). However, the parallel responses of grazers and producers to the nutrient addition treatment could not be confirmed (H_1_). For zooplankton diversity, the expectation that a metacommunity structure would intensify the positive effects of pulsed nutrient addition (H_2_) was not only confirmed, but also appeared to be conditional for the species survival. We could clearly observe the negative interactive effects of pulsed nutrients and isolated communities, with the lowest grazer diversity values in IP treatments (Fig. 1a). However, the positive effect of MP treatments was time dependent (Tables 1 and 2), with transient higher local and regional diversities appearing between the second and fourth week (Fig. 1a, c). Moreover, measures of grazers’ diversity differed significantly between scales (Fig. 1a, c), in contrast to the autotrophs’ diversity that showed nearly the same responses at both scales (Fig. 1b, d). We could observe a longer persistence of zooplankton diversity at the regional scale, with slight differences between pulsed and continuous nutrient additions. In the MC treatment, grazer diversity dropped during the middle of the experiment, however recovering subsequently. This instability was not observed in MP treatments, which had less variation, even though there was a slight decrease in Shannon diversity from the third week on (Fig. 1c).

**Table 1:** Two-way ANOVA with repeated measures

	Landscape		Nutrient		Time		Landscape: Nutrient		Landscape: Time		Nutrient: Time	
	*F*_1,8_	*p*	*F*_1,8_	*p*	*F*_6,48_	*p*	*F*_1,8_	*p*	*F*_6,48_	*p*	*F*_6,48_	*p*
Shannon (zoo)	1.036	0.338	0.894	0.372	25.36	**< 0.001**	5.082	**0.028**	1.481	0.205	1.405	0.232
Biomass (zoo)	26.584	**< 0.001**	6.355	**0.035**	10.60	**< 0.001**	10.19	**0.012**	11.62	**< 0.001**	0.941	0.167
Shannon (phyto)	1.474	0.259	16.37	**0.003**	37.45	**< 0.001**	0.051	0.827	2.504	**0.034**	3.748	**0.003**
Biomass (phyto)	31.59	**< 0.001**	25.55	**< 0.001**	131	**< 0.001**	7.75	**0.023**	0.134	0.724	20.931	**0.002**

Testing the effects of landscape, nutrient addition and time on Shannon diversity and biomass of the zooplankton (zoo) and phytoplankton (phyto) communities at the local scale. Significant *p* values are in bold

*Shannon = Shannon–Wiener diversity

**Table 2:** One-way ANOVA with repeated measures

	Nutrient		Time		Nutrient: Time	
	*F*_1,10_	*p*	*F*_6,60_	*p*	*F*_6,60_	*p*
Shannon (zoo)	0.638	0.443	13.15	**< 0.001**	1.375	0.239
Biomass (zoo)	1.195	0.03	6.325	**< 0.001**	0.737	0.599
Shannon (phyto)	16.05	**0.002**	27.23	**< 0.001**	3.053	**0.011**
Biomass (phyto)	3.344	0.141	56.45	**0.002**	8.068	**0.047**

Testing the effects nutrient addition and time on Shannon diversity and biomass of the zooplankton (zoo) and phytoplankton (phyto) communities at the regional scale. Significant *p* values are in bold

*Shannon = Shannon–Wiener diversity

The analyses of species richness and evenness are shown only in the Electronic supplementary material, since it presented similar patterns to Shannon index results. However, it is important to highlight that the higher phytoplankton diversity under pulsed nutrient addition was mainly driven by higher species evenness. In addition, the higher number of autotrophs species was kept longer in a dependency of the metacommunity landscape (MP). For zooplankton Shannon diversity, the respectively positive and negative relationships of pulsed nutrient addition with metacommunity and isolated community were mainly shaped by richness [see Online Resource Fig. S1 and S2, Table S1 and S2 for richness and evenness results].

### Dissimilarity among metacommunity patches and the ß-diversity components

**Fig. 2:**
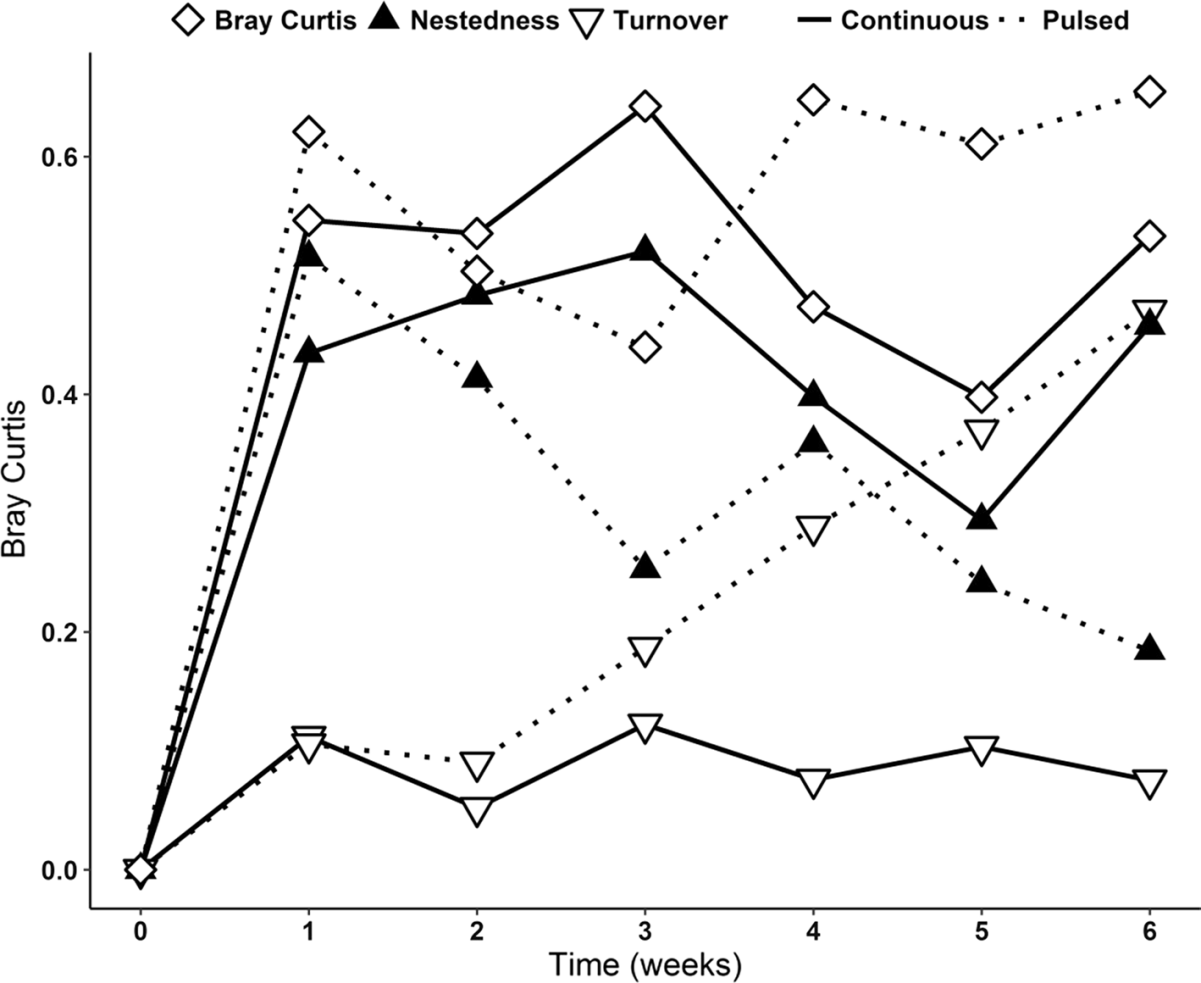
Partitioning of beta-diversity. The beta-diversity was calculated as Bray–Curtis multiple-site dissimilarities based on the local abundance data from zooplankton and phytoplankton computed for 6 weeks of experiment. Total beta-diversity, measured as Bray–Curtis distances, is represented by the open diamonds. Nestedness is represented by the filled triangles and turnover is represented by the open triangles

Interestingly, the patches of a metacommunity developed differently over time, even with equally applied nutrient additions in the four patches. The degree of dissimilarity among sites varied over time in both metacommunities, showing a strong influence of nutrient addition in promoting temporal spatial heterogeneity (Table 3). Beta-diversity (measured as Bray–Curtis distances) increased significantly under both pulsed and continuous treatments (Fig. 2) after 1 week of the experiment. From that point on, the nutrient addition started to significantly influence the species organization among the patches. Corroborating our assumptions (H_3_), higher beta-diversity was fostered by pulsed nutrient addition, indicating that the MP patches became more dissimilar in species abundance than the MC patches, but only from the middle of the experiment onwards.

**Table 3:** One-way ANOVA with repeated measures

	Nutrient		Time		Nutrient: Time	
	*F*_1,24_	*p*	*F*_5,24_	*p*	*F*_5,24_	*p*
Beta-diversity	16.99	**< 0.001**	9.23	**< 0.001**	0.381	0.85

Testing the effects of beta-diversity on metacommunity landscape. The data refer to zooplankton and phytoplankton together. Significant *p* values are in bold

Considerable differences in the source of dissimilarity were encountered between the two different nutrient additions (Fig. 2). Nestedness (abundance gradients) was the most important factor influencing beta-diversity in metacommunities with continuous nutrient supply during the 6 weeks of experiment. Nestedness also influenced beta-diversity in MP treatments at the initial phase of the experiment, but its significance was not persistent. From the second week on, turnover started to take place, resulting in a remarkable increase of beta-diversity in these metacommunities.

### Species biomass and resource use efficiency (RUE)

**Fig. 3:**
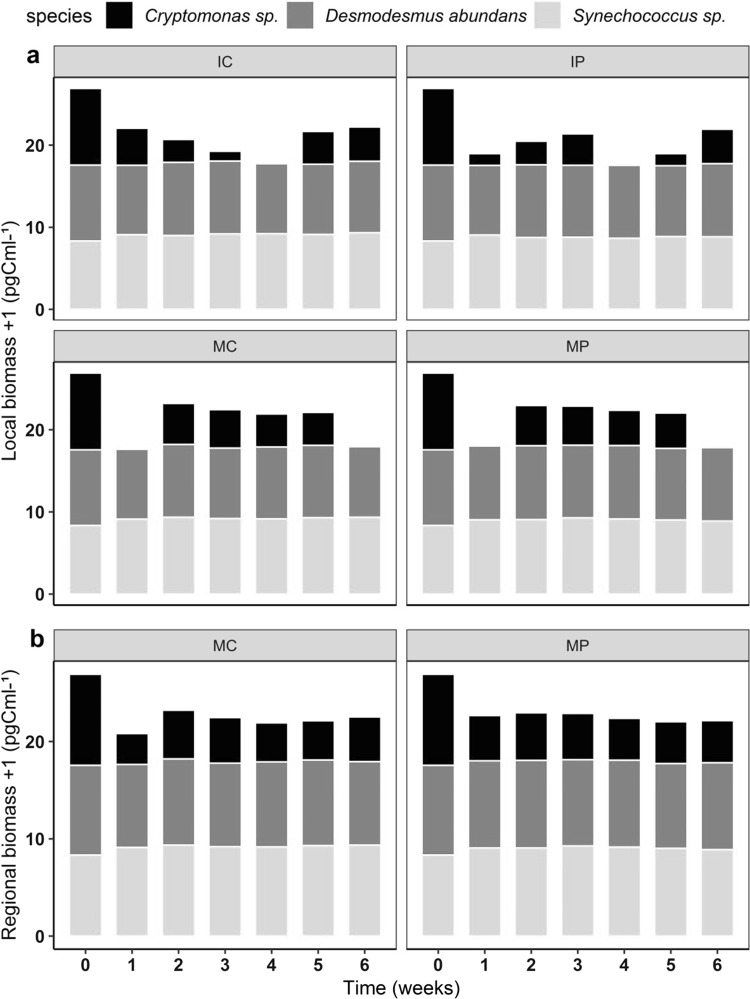
Absolute biomass of the three species of phytoplankton. **a** Local biomass; **b** regional biomass. IC isolated community with continuous nutrient addition, IP isolated community with pulsed nutrient addition, MC metacommunity with continuous nutrient addition, MP metacommunity with pulsed nutrient addition. Values are mean ± SE, *n* = 3. Note log scale used in panel (log_10_ + 1). A color version of the figure is available online

The biomass of both groups of organisms was significantly affected by the interactive effects of landscape and nutrient addition (Table 1). The biomass of phytoplankton varied over time among the four treatment combinations, mainly because of fluctuations in *Cryptomonas sp*. biomass (Fig. 3a). It was possible to observe the rarity of this species in all four treatments, but most in isolated communities with pulsed nutrient addition (Online Resource Fig. S3a). The higher biomass (Fig. 3) in metacommunities than in isolated communities shows the strong influence of the landscape on the local phytoplankton biomass (Table 1; *F*_1,8_ = 31.59; *p* < 0.001). Nutrient addition also affected this species’ biomass, and the influence was time dependent (Table 1; *F*_6,48_ = 20.931; *p* = 0.002). In isolated communities, pulsed supply showed lower absolute biomass in comparison to the continuous treatment. In metacommunities, the pulsed treatment sustained the highest abundance of *Cryptomonas sp*. over all treatments, and the highest absolute biomass, which was mostly contributed by *Desmodesmus abundans* (Online Resource Fig. S3c). In contrast, *Synechococcus sp.* was the dominating species in MC treatments, showing a strong increase in abundance after the fourth week (Online Resource Fig. S3b). At regional scale, the effect of nutrient on phytoplankton biomass was less significant (Fig. 3b; Table 2) than locally; however, both scales showed similar patterns, with highest absolute biomass in metacommunities with pulsed nutrient addition. This effect remained only until the fourth week, which can be related to the strong decrease in the *Synechococcus sp.* abundance (Online Resource Fig. S3b).

**Fig. 4:**
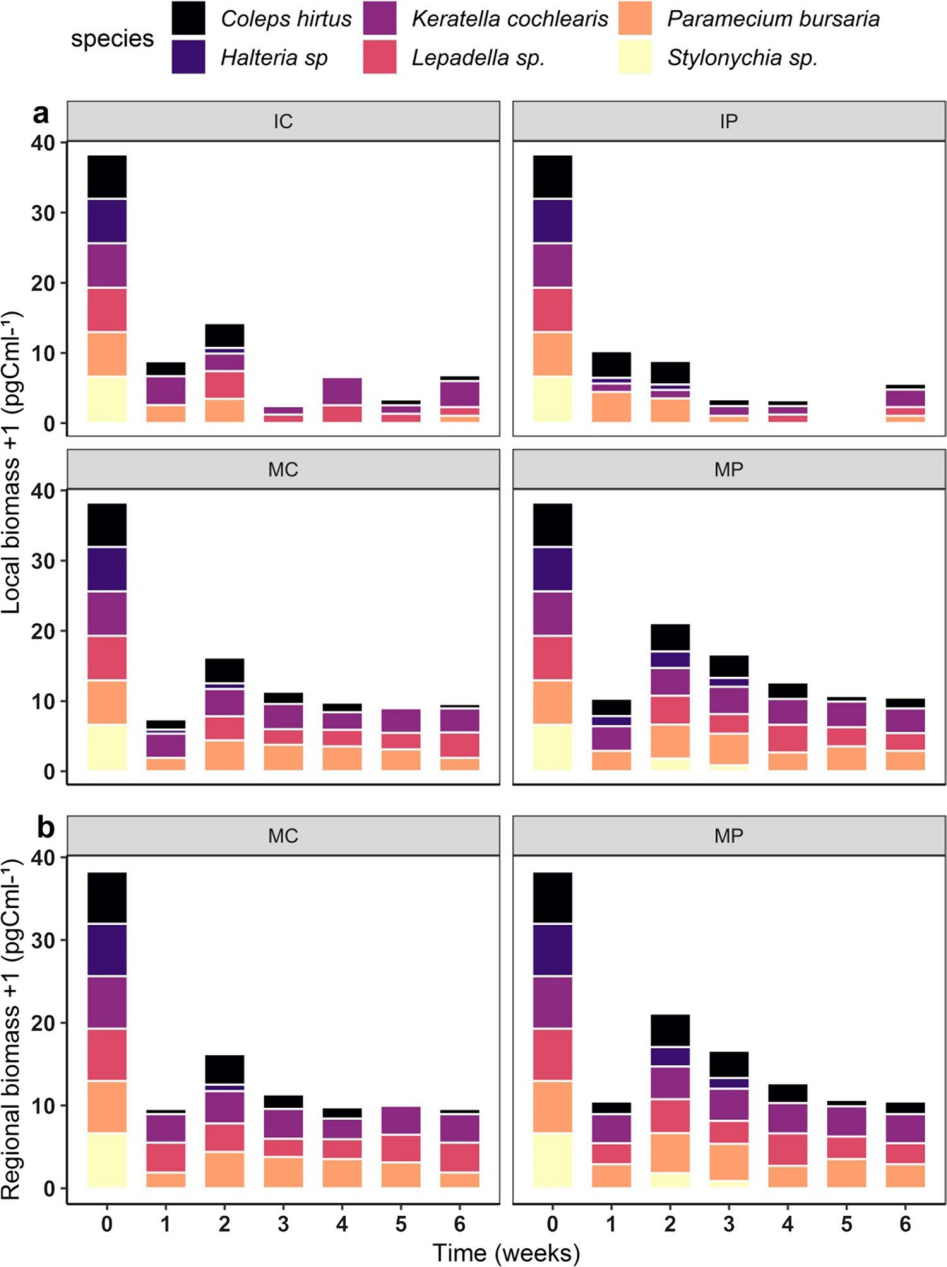
Absolute biomass of the six species of zooplankton. **a** Local biomass; **b** regional biomass. IC isolated community with continuous nutrient addition, IP isolated community with pulsed nutrient addition, MC metacommunity with continuous nutrient addition, MP metacommunity with pulsed nutrient addition. Values are mean ± SE, *n* = 3. Note log scale used in panel (log_10_ + 1). A color version of the figure is available online

Landscape was the factor that most affected zooplankton biomass, with the metacommunity landscape sustaining the highest absolute biomass over time (Fig. 4). This positive effect of interconnected communities was consistent over time, as well as the effect of the interaction between landscape and nutrient addition (Table 1). IP and MP represent the treatments with lowest and highest absolute biomass, respectively. This result shows the landscape dependency of the nutrient addition effect, and Tukey’s post hoc tests revealed that biomass was positively influenced by a metacommunity landscape combined with pulsed nutrient addition. Conversely, in isolated communities, pulsing nutrients had a negative effect on the grazers’ biomass (Fig. 4a). In both local and regional scales, we could observe higher biomass in metacommunities with pulsed nutrient addition. Three species constantly contributed most to the absolute biomass in the MP treatment: *Keratella cochlearis, Paramecium bursaria* and *Lepadella sp.* Another ciliate, *Coleps hirtus*, also contributed to the biomass, but with a decreasing trend. *Halteria sp.* and *Stylonychia sp.* had very low abundances (Fig. 4), disappearing from the fourth week on in the MP treatment. These two ciliates disappeared earlier in the other three treatments (IC, IP and MC), *Stylonychia sp.* from the first week on and *Halteria sp.* from the second week on (Fig. 4 and Online Resource Fig. S4). In isolated communities, the effect of nutrient addition was the opposite of what we found in metacommunities, with highest absolute biomass sustained by continuous nutrient addition. In the isolated landscape, the rotifer *Keratella cochlearis* was the dominating species, contributing most to the biomass in both isolated communities, but persisting longer in the IC than in the IP treatment. Analyzing the dynamics of each of the grazer groups separately, the responses of ciliates and rotifers were different. The former had a remarkable increase in abundance, mostly in metacommunities at the initial phase of the experiment, showing a strong decline from the second week on in all treatments (Online Resource Fig. S4a, b, e, and f). Rotifers showed lower abundances in comparison to the ciliates in metacommunities, and they persisted longer in isolated communities (Online Resource Fig. S4c, d).

**Fig. 5:**
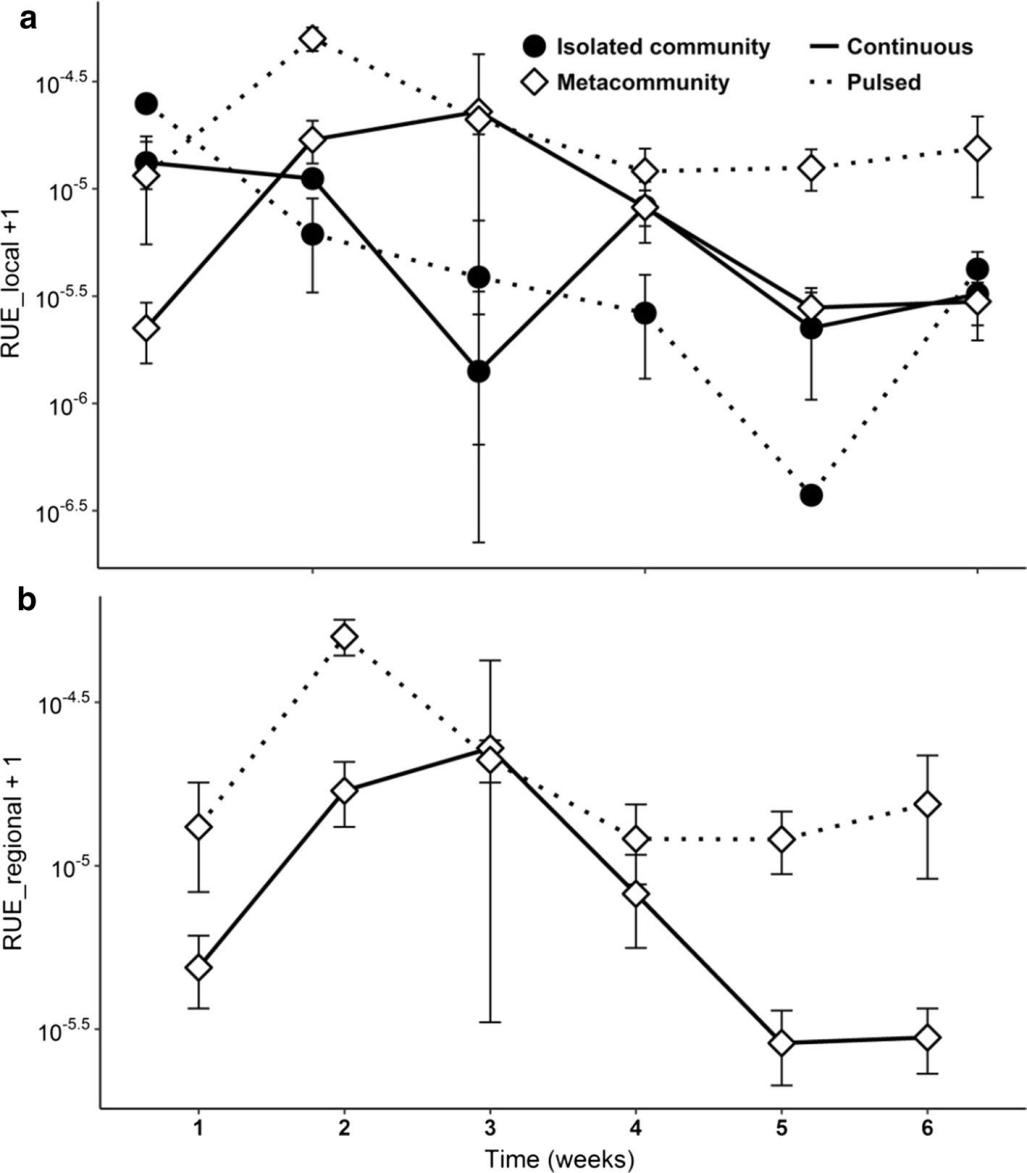
Resource use efficiency (RUE) over time. RUE measured as zooplankton biomass per unit phytoplankton biomass. Local RUE (**a**) was estimated for metacommunities and isolated communities. Regional RUE (**b**) was estimated only for metacommunities. Values are mean ± SE, *n* = 3. Note log scale used in panel (log_10_ + 1)

Resource use efficiency (RUE), measured as the ratio between zooplankton and phytoplankton biomass, showed strong differences among the four treatments (Fig. 5). In both isolated communities, the ratio decreased from the first week onwards (Fig. 5a), except for the slightly increase on the third week in the IC treatments. Furthermore, the RUE decrease was faster in the IP treatments than in the IC treatments. In metacommunities, differences between local and regional scale were not considerable. Differently from isolated communities, both metacommunities showed an initial increase in RUE. While metacommunities with continuous nutrient addition had a remarkable decrease in RUE after the third week, at the same time the ratio stabilized in the MP treatments. Additionally, the latter treatment supported the highest values of RUE over time, in agreement with our assumption (H_4_), of the lowest RUE promoted by the IP treatments.

## Discussion

The questions addressed in this study concerning metacommunity dynamics and how they interact with eutrophication in influencing biodiversity are subjects that cannot be easily approached at a macroscopic scale. The aquatic model system used could be experimentally manipulated on a reasonable timeframe, allowing the simulation of human impacts with a microcosm design. Additionally, the individuals used as model organisms co-occur in nature and showed sensitivity to the treatments tested, in agreement with previous work that successfully used protists to illuminate several aspects of metacommunity dynamics (Limberger and Wickham 2011a, b, 2012a, b). The two factors manipulated here, nutrient addition and landscape, showed direct positive or negative effects depending on the group of organisms analyzed, as well as significant interactive effects. Nutrient addition was the treatment that affected phytoplankton diversity most, with a persistent positive effect of pulsed supply, consistent with previous studies (Sommer 1985; Flöder and Sommer 1999). Additionally, our results show that the same response in phytoplankton can be seen independently of landscape type and under grazing pressure, even at limited phytoplankton species richness. Partially rejecting H_1_, we could not confirm a parallel response of grazers and producers under pulsed nutrient addition, as we found very low grazer diversity and very high producer diversity in IP treatments. The very low grazer diversity in IP treatments is explained by the quality of prey that was dominating this habitat. We used three prey species with different sizes and nutritional qualities, which could have influenced the generalists’ consumption and growth. Despite the positive results for autotrophs in IP treatments, the abundance of the flagellate *Cryptomonas sp*. was close to zero over the entire experiment, becoming nearly unavailable as a food resource from the first week on in this treatment. It has been widely suggested that *Cryptomonas sp*. is a preferred food source of zooplankton (Stemberger 1981; Skogstad et al. 1987; Ahlgren et al. 1990; Demott and Müller-navarra 1997) and this could have been the case in our experiment as well. Moreover, it is known that changes in nutrient availability can drastically affect the consumers through their prey composition (Laspoumaderes et al. 2010; Glibert et al. 2013). In our microcosms, the phytoplankton biomass was mostly contributed by the green algae *Desmodesmus abundans,* together with *Synechococcus sp*. (in all four treatment combinations). Cyanobacteria are commonly classified as a poor food source for zooplankton because of their deficiency in essential omega-3 fatty acids, whereas green algae are of better food quality, except for their relatively low concentrations of highly unsaturated fatty acids (Taipale et al. 2016; Peltomaa et al. 2017). Furthermore, it has been shown that *Desmodesmus* forms colonies against the grazing pressure, which can hamper zooplankton growth (Vanormelingen et al. 2009). Cryptomonads, however, are rated as high food quality because of their rich lipid composition (Skogstad et al. 1987; Demott and Müller-navarra 1997; Marzetz et al. 2017; Taipale et al. 2018). In previous studies, grazer growth was suggested to increase with fatty acid supply encountered in primary producers (Demott and Müller-navarra 1997; Marzetz et al. 2017). This implies, however, that high phytoplankton diversity and biomass are of less importance than the abundance of high quality prey: in the isolated communities with pulsed nutrient addition, the low *Cryptomonas* sp. abundance also meant that the average quality of available food was relatively low, resulting in a very low diversity and biomass of zooplankton. Conversely, in the MP treatment, where the phytoplankton diversity was also higher, the grazers could find more favorable conditions, due to the higher abundance of *Cryptomonas sp*. in this treatment, in combination with an interconnected landscape. Supporting this idea, a previous study has demonstrated the importance of high food quality (cryptophytes), even in low abundances, for the nutritional support of zooplankton (Taipale et al. 2018). Therefore, our second hypothesis (H_2_) was confirmed only for the zooplankton community, with higher diversity being observed in MP treatments. However, and partially contradicting H_2_, landscape had little effect on phytoplankton diversity. This result might not be surprising, since only one species was actively motile. However, we expected grazer responses to reflect on their prey, in a cascading top-down control (H_2_′). Nonetheless, consistent with a recent study (Gusha et al. 2019), our results indicate that the bottom-up effects were more significant than the inverse relation in controlling the phytoplankton species. Furthermore, previous studies have demonstrated that with increasing trophic levels, the sensitivity to landscape conditions also increases (Sergio et al. 2008; Blüthgen et al. 2016). This can be related to the general assumption that larger organisms are more mobile than smaller organisms (Peters 1986) and, therefore, a wider spatial scale can be covered by the former, while the latter tend to remain more locally (McCann et al. 2005; McCann and Rooney 2009). Because of the larger body size, the larger area occupied and the lower abundance, these organisms are more affected by habitat fragmentation (Holyoak 2000b; Duffy 2003; Staddon et al. 2010). The overall greater impact of metacommunities on grazers traits can be related broadly to results showing the relevance of dispersal for the maintenance of ecosystem functions (Loreau et al. 2003; Gonzalez and Loreau 2009; Verreydt et al. 2012). Moreover, the dispersal of species among local communities is long known to be a key of species persistence at regional scale, preventing biodiversity losses (Andrewartha and Birch 1986; Holyoak et al. 2005; Howeth and Leibold 2010). Our results suggest that not only an interconnected landscape is relevant for regional species survival, but also that good quality food in at least one patch is important in maintaining grazer diversity.

No less important than local and regional diversity, beta-diversity and its components are valuable in understanding metacommunity organization and dynamics, and are important issues for conservation management (Soininen et al. 2007; Heino 2011; Suurkuukka et al. 2012). Habitat heterogeneity is suggested to be positively correlated with beta-diversity (Gabriel et al. 2006; Patzkowsky and Holland 2007; Suurkuukka et al. 2012). Interestingly, even under equal treatment conditions, the four patches of a metacommunity showed increased dissimilarities in species abundance over time, but to a greater degree under pulsed nutrient supply. Thus, we could confirm our assumption that pulsed supply promotes higher stochasticity and, therefore, higher dissimilarity among patches (H_3_). Various nutritional niches can be formed as a consequence of the different degrees of nutrient availability, which enable a higher number of species to coexist according to their survival abilities (Chesson 2000; Behmer and Joern 2008; Roelke and Spatharis 2015).

While the increase in beta-diversity was primarily due to nestedness in both metacommunities, it decreased in importance over time in MP treatments, being superseded by turnover. Nestedness was the dominating component of beta-diversity in MC treatments, reflecting that with continuous nutrient addition one patch was species-rich while other patches were species-poor, which in turn indicates differences in species abundance gradients among sites. The existence of only few patches supporting higher number of species (Patterson 1987) could be the reason why beta-diversity has been negatively related to nestedness (Wright and Reeves 1992). In a different way, the MP treatments had the turnover component scaling up from the second week on, while nestedness decreased. It has been suggested that beta-diversity shaped by the replacement of species (turnover) can indicate the predominance of the species-sorting process (Legendre and Cáceres 2013; Yang et al. 2018), which implies that environmental heterogeneity was an important factor in structuring this metacommunity type (MP). A previous study has also suggested a positive relationship between species replacement and habitat heterogeneity when dispersal is possible (i.e., in a metacommunity) (Gianuca et al. 2017). Once the weekly nutrient supply created temporal differences in nutrient concentrations among the metacommunity patches, it also fostered higher dissimilarities in community compositions. The prevalence of nestedness in MC treatments contradicts the results from a recent meta-analysis of beta-diversity components, which concluded that most of the studies have found turnover as the main driver of beta-diversity (Soininen et al. 2018). Therefore, our study provides interesting results of nestedness and turnover, which were found to be strongly influenced by the temporal differences in nutrient addition.

We assumed that higher RUE would be a consequence of the MP treatment (H4), since the resource use efficiency of an ecosystem is expected to be positively correlated with the number of species coexisting in the environment (Tilman et al. 1997; Abonyi et al. 2018). In agreement with our assumption, the RUE in both metacommunities showed an increase after the first week, but declining subsequently. Nonetheless, the decrease in MP treatments was slower than in MC treatments, stabilizing by the fourth week. In the isolated communities, we observed a persistent decrease of the biomass ratio between zooplankton and phytoplankton, which can be related to a declining number of grazers found in this landscape, rather than an increase in phytoplankton.

In summary, landscape structure and nutrient addition influenced plankton diversity, with different patterns for primary producers and grazers. The benefit of a metacommunity structure, with an appropriate interconnection among patches, was confirmed in our study, but mainly for grazers’ diversity. Nutrient supply was also important for species organization, directly affecting the primary producers. The interaction between the two treatments was extremely important for grazer survival. The Shannon index can potentially lose information by combining aspects of richness and evenness. In our study, however, Shannon diversity, when combined with abundance data, well described the species dynamics in the experimental treatments (Spatharis et al. 2011). Furthermore, the highest beta-diversity was promoted by pulsed nutrient addition, indicating that MP treatment resulted in greater patch dissimilarity and in greater balance in species abundance among the sites (higher turnover). Our results suggest that if the time between nutrient pulses is sufficient, species coexistence can be facilitated in a metacommunity landscape even when nutrient addition is simultaneous in all patches. Finally, all statistical analyses showed strong time dependency, as has been seen in previous work using different aquatic model communities (Limberger and Wickham 2012b), indicating how important it is to take into account the temporal aspects of the experiments.

The results achieved with this work and their interpretation can only aim at a very small slice of the interaction between metacommunities and nutrient addition. Clearly, there is still plenty of interesting work to be done. There are many more possibilities of testing anthropogenic factors on a microcosm scale, and if the results are carefully interpreted, they can contribute to a better understanding of biodiversity loss in real scenarios.

## Electronic supplementary material

Below is the link to the electronic supplementary material.

Supplementary material 1 (DOC 8177 kb)

## References

[CR1] Abonyi A, Horváth Z, Ptacnik R (2018). Functional richness outperforms taxonomic richness in predicting ecosystem functioning in natural phytoplankton communities. Freshw Biol.

[CR2] Ahlgren G, Lundstedt L, Brett M, Forsberg C (1990). Lipid composition and food quality of some freshwater phytoplankton for cladoceran zooplankters. J Plankton Res.

[CR3] Andrewartha HG, Birch LC (1986). The ecological web: more on the distribution and abundance of animals.

[CR4] Angeler DG (2013). Revealing a conservation challenge through partitioned long-term beta diversity: increasing turnover and decreasing nestedness of boreal lake metacommunities. Divers Distrib.

[CR5] Baselga A (2010). Partitioning the turnover and nestedness components of beta diversity. Glob Ecol Biogeogr.

[CR6] Baselga A (2013). Separating the two components of abundance-based dissimilarity: balanced changes in abundance vs. abundance gradients. Methods Ecol Evol.

[CR7] Baselga A (2017). Partitioning abundance-based multiple-site dissimilarity into components: balanced variation in abundance and abundance gradients. Methods Ecol Evol.

[CR8] Baselga A, Orme CDL (2012). betapart: an R package for the study of beta diversity. Methods Ecol Evol.

[CR9] Behmer ST, Joern A (2008). Coexisting generalist herbivores occupy unique nutritional feeding niches. Proc Natl Acad Sci.

[CR10] Blüthgen N, Simons NK, Jung K, Prati D, Renner SC, Boch S (2016). Land use imperils plant and animal community stability through changes in asynchrony rather than diversity. Nat Commun.

[CR11] Cardinale BJ (2011). Biodiversity improves water quality through niche partitioning. Nature.

[CR12] Chase JM, Leibold MA (2002). Spatial scale dictates the productivity-biodiversity relationship. Nature.

[CR13] Chesson P (2000). General theory of competitive coexistence in spatially-varying environments. Theor Popul Biol.

[CR14] Chisholm C, Lindo Z, Gonzalez A (2011). Metacommunity diversity depends on connectivity and patch arrangement in heterogeneous habitat networks. Ecography.

[CR15] Cook SC, Housley L, Back JA, King RS (2018). Freshwater eutrophication drives sharp reductions in temporal beta diversity. Ecology.

[CR16] de Mazancourt C, Isbell F, Larocque A, Berendse F, De Luca E, Grace JB (2013). Predicting ecosystem stability from community composition and biodiversity. Ecol Lett.

[CR17] Demott W, Müller-navarra D (1997). The importance of highly unsaturated fatty acids in zooplankton nutrition: evidence from experiments with Daphnia, a cyanobacterium and lipid emulsions. Freshw Biol.

[CR18] Duffy JE (2003). Biodiversity loss, trophic skew and ecosystem functioning. Ecol Lett.

[CR19] Flöder S, Sommer U (1999). Diversity in planktonic communities: an experimental test of the intermediate disturbance hypothesis. Limnol Oceanogr.

[CR20] Gabriel D, Roschewitz I, Tscharntke T, Thies C (2006). Beta diversity at different spatial scales: plant communities in organic and conventional agriculture. Ecol Appl.

[CR21] Gianuca AT, Declerck SA, Lemmens P, De Meester L (2017). Effects of dispersal and environmental heterogeneity on the replacement and nestedness components of β-diversity. Ecology.

[CR22] Gibson L, Lynam AJ, Bradshaw CJA, He F, Bickford DP, Woodruff DS (2013). Near-complete extinction of native small mammal fauna 25 years after forest fragmentation. Science.

[CR23] Glibert PM, Kana TM, Brown K (2013). From limitation to excess: the consequences of substrate excess and stoichiometry for phytoplankton physiology, trophodynamics and biogeochemistry, and the implications for modeling. J Mar Syst.

[CR24] Gonzalez A, Loreau M (2009). The causes and consequences of compensatory dynamics in ecological communities. Annu Rev Ecol Evol Syst.

[CR25] Goslee SC, Urban DL (2007). The ecodist package for dissimilarity-based analysis of ecological data. J Stat Softw.

[CR26] Gusha MNC, Dalu T, Wasserman RJ, McQuaid CD (2019). Zooplankton grazing pressure is insufficient for primary producer control under elevated warming and nutrient levels. Sci Total Environ.

[CR27] Heino J (2011). A macroecological perspective of diversity patterns in the freshwater realm. Freshw Biol.

[CR28] Heino J (2013). The importance of metacommunity ecology for environmental assessment research in the freshwater realm. Biol Rev.

[CR29] Hillebrand H, Lehmpfuhl V (2011). Resource stoichiometry and consumers control the biodiversity-productivity relationship in pelagic metacommunities. Am Nat.

[CR30] Holyoak M (2000). Habitat patch arrangement and metapopulation persistence of predators and prey. Am Nat.

[CR31] Holyoak M (2000). Habitat subdivision causes changes in food web structure. Ecol Lett.

[CR32] Holyoak M, Lawler SP (1996). Persistence of an extinction-prone predator-prey interaction through metapopulation dynamics. Ecol.

[CR33] Holyoak M, Leibold MA, Holt RD (2005). Metacommunities: spatial dynamics and ecological communities.

[CR34] Hothorn T, Bretz F, Westfall P (2008). Simultaneous inference in general parametric models. Biom J.

[CR35] Howeth JG, Leibold MA (2010). Species dispersal rates alter diversity and ecosystem stability in pond metacommunities. Ecology.

[CR36] Kassen R, Buckling A, Bell G, Rainey PB (2000). Diversity peaks at intermediate productivity in a laboratory microcosm. Nature.

[CR37] Laspoumaderes C, Modenutti B, Balseiro E (2010). Herbivory versus omnivory: linking homeostasis and elemental imbalance in copepod development. J Plankton Res.

[CR38] Legendre P, Cáceres M (2013). Beta diversity as the variance of community data: dissimilarity coefficients and partitioning. Ecol Lett.

[CR39] Lenth RV (2016). Least-squares means: the R package lsmeans. J Stat Softw.

[CR40] Limberger R, Wickham SA (2011). Competition–colonization trade-offs in a ciliate model community. Oecologia.

[CR41] Limberger R, Wickham SA (2011). Predator dispersal determines the effect of connectivity on prey diversity. PLoS One.

[CR42] Limberger R, Wickham SA (2012). Disturbance and diversity at two spatial scales. Oecologia.

[CR43] Limberger R, Wickham SA (2012). Transitory versus persistent effects of connectivity in environmentally homogeneous metacommunities. PLoS One.

[CR44] Loreau M, Mouquet N, Gonzalez A (2003). Biodiversity as spatial insurance in heterogeneous landscapes. Proc Natl Acad Sci.

[CR45] MacArthur RH, Wilson EO (1967). The theory of Island biogeography.

[CR46] Marzetz V, Koussoroplis A-M, Martin-Creuzburg D, Striebel M, Wacker A (2017). Linking primary producer diversity and food quality effects on herbivores: a biochemical perspective. Sci Rep.

[CR47] McCann KS, Rooney N (2009). The more food webs change, the more they stay the same. Philos Trans R Soc B Biol Sci.

[CR48] McCann KS, Rasmussen J, Umbanhowar J (2005). The dynamics of spatially coupled food webs. Ecol Lett.

[CR49] Oksanen J, Blanchet FG, Friendly M, Kindt R, Legendre P, McGlinn D, Minchin PR, O'Hara RB, Simpson GL, Solymos P et al (2017) vegan: Community Ecology Package. R package version 2.4-5

[CR50] Papanikolopoulou LA, Smeti E, Roelke DL, Dimitrakopoulos PG, Kokkoris GD, Danielidis DB (2018). Interplay between* r*-and* K*-strategists leads to phytoplankton underyielding under pulsed resource supply. Oecologia.

[CR51] Patterson BD (1987). The principle of nested subsets and its implications for biological conservation. Conserv Biol.

[CR52] Patzkowsky ME, Holland SM (2007). Diversity partitioning of a Late Ordovician marine biotic invasion: controls on diversity in regional ecosystems. Paleobiology.

[CR53] Peltomaa ET, Aalto SL, Vuorio K, Taipale SJ (2017). The importance of phytoplankton biomolecule availability for secondary production. Front Ecol Evol.

[CR54] Peters RH (1986). The ecological implications of body size.

[CR55] Pinheiro J (2018) nlme: Linear and nonlinear mixed effects models. http://cran.r-project.org/web/packages/nlme/index.html

[CR56] Putt M, Stoecker DK (1989). An experimentally determined carbon: volume ratio for marine “oligotrichous” ciliates from estuarine and coastal waters. Limnol Oceanogr.

[CR57] Redfield AC (1958). The biological control of chemical factors in the environment. Am Sci.

[CR58] Resetarits EJ, Cathey SE, Leibold MA (2018). Testing the keystone community concept: effects of landscape, patch removal, and environment on metacommunity structure. Ecology.

[CR59] Rocha O, Duncan A (1985). The relationship between cell carbon and cell volume in freshwater algal species used in zooplanktonic studies. J Plankton Res.

[CR60] Roelke D (2000). Copepod food-quality threshold as a mechanism influencing phytoplankton succession and accumulation of biomass, and secondary productivity: a modeling study with management implications. Ecol Model.

[CR61] Roelke DL, Eldridge PM (2007). Mixing of supersaturated assemblages and the precipitous loss of species. Am Nat.

[CR62] Roelke DL, Spatharis S (2015). Phytoplankton succession in recurrently fluctuating environments. PLoS One.

[CR63] Sergio F, Caro T, Brown D, Clucas B, Hunter J, Ketchum J (2008). Top predators as conservation tools: ecological rationale, assumptions, and efficacy. Annu Rev Ecol Evol Syst.

[CR64] Skogstad A, Granskog L, Klaveness D (1987). Growth of freshwater ciliates offered planktonic algae as food. J Plankton Res.

[CR65] Smeti E, Roelke DL, Spatharis S (2016). Spatial averaging and disturbance lead to high productivity in aquatic metacommunities. Oikos.

[CR66] Soininen J, Lennon JJ, Hillebrand H (2007). A multivariate analysis of beta diversity across organisms and environments. Ecology.

[CR67] Soininen J, Heino J, Wang J (2018). A meta-analysis of nestedness and turnover components of beta diversity across organisms and ecosystems. Glob Ecol Biogeogr.

[CR68] Sommer U (1985). Comparison between steady state and non-steady state competition: experiments with natural phytoplankton. Limnol Oceanogr.

[CR69] Spatharis S, Roelke DL, Dimitrakopoulos PG, Kokkoris GD (2011). Analyzing the (mis) behavior of Shannon index in eutrophication studies using field and simulated phytoplankton assemblages. Ecol Ind.

[CR70] Staddon P, Lindo Z, Crittenden PD, Gilbert F, Gonzalez A (2010). Connectivity, non-random extinction and ecosystem function in experimental metacommunities. Ecol Lett.

[CR71] Steiner CF, Stockwell RD, Kalaimani V, Aqel Z (2013). Population synchrony and stability in environmentally forced metacommunities. Oikos.

[CR72] Stemberger RS (1981). A general approach to the culture of planktonic rotifers. Can J Fish Aquat Sci.

[CR73] Stemberger RS, Gilbert JJ (1987). Rotifer threshold food concentrations and the size-efficiency hypothesis. Ecology.

[CR74] Suurkuukka H, Meissner KK, Muotka T (2012). Species turnover in lake littorals: spatial and temporal variation of benthic macroinvertebrate diversity and community composition. Divers Distrib.

[CR75] Taipale SJ, Hiltunen M, Vuorio K, Peltomaa E (2016). Suitability of phytosterols alongside fatty acids as chemotaxonomic biomarkers for phytoplankton. Front Plant Sci.

[CR76] Taipale SJ, Kahilainen KK, Holtgrieve GW, Peltomaa ET (2018). Simulated eutrophication and browning alters zooplankton nutritional quality and determines juvenile fish growth and survival. Ecol Evol.

[CR77] Taylor JM, King RS, Pease AA, Winemiller KO (2014). Nonlinear response of stream ecosystem structure to low-level phosphorus enrichment. Freshw Biol.

[CR78] Tilman D, Downing JA (1994). Biodiversity and stability in grasslands. Nature.

[CR79] Tilman D, Lehman CL, Thomson KT (1997). Plant diversity and ecosystem productivity: theoretical considerations. Proc Natl Acad Sci USA.

[CR80] Tilman D, Reich PB, Knops J, Wedin D, Mielke T, Lehman C (2001). Diversity and productivity in a long-term grassland experiment. Science.

[CR81] Ulrich W, Gotelli NJ (2007). Null model analysis of species nestedness patterns. Ecology.

[CR82] Vanormelingen P, Vyverman W, De Bock D, Van der Gucht K, Meester LD (2009). Local genetic adaptation to grazing pressure of the green alga *Desmodesmus armatus* in a strongly connected pond system. Limnol Oceanogr.

[CR83] Venables WN, Ripley BD (2013). Modern applied statistics with S-PLUS.

[CR84] Verreydt D, De Meester L, Decaestecker E, Villena MJ, Van Der Gucht K, Vannormelingen P (2012). Dispersal-mediated trophic interactions can generate apparent patterns of dispersal limitation in aquatic metacommunities. Ecol Lett.

[CR85] Wickham H, Francois R (2016) dplyr: a grammar of data manipulation. R package version 0.5.0

[CR86] Wright DH, Reeves JH (1992). On the meaning and measurement of nestedness of species assemblages. Oecologia.

[CR87] Yang Y, Hu R, Lin Q, Hou J, Liu Y, Han B-P (2018). Spatial structure and β-diversity of phytoplankton in Tibetan Plateau lakes: nestedness or replacement?. Hydrobiologia.

