# Supplementary material for: Simulating eutrophication in a metacommunity landscape: an aquatic model ecosystem
Source: Oecologia. 2018 Dec 6;189(2):461–74. doi: 10.1007/s00442-018-4319-8 (PMC6394664; doi:10.1007/s00442-018-4319-8)
Supplement: Supplementary file 1 — Supplementary material 1 (DOC 8177 kb) [file 442_2018_4319_MOESM1_ESM.doc]

Electronic Supplemental Material for **“**Simulating eutrophication in a metacommunity landscape – an aquatic model ecosystem**”**

Josie Antonucci di Carvalho and Stephen A. Wickham

correspondence: [josie.carvalho @stud.sbg.ac.at](mailto:romana.limberger@stud.sbg.ac.at)

**Table S1** Two way ANOVA with repeated measures. Testing the effects of landscape, nutrient addition and time on Shannon diversity, richness, evenness and biomass of the **zooplankton (zoo)** and **phytoplankton** **(phyto)** communities at the **local scale**. Significant p - values are in bold

| **Zoo** | **Landscape** | | **Nutrient** | | **Time** | | **Landscape: Nutrient** | | **Landscape:**  **Time** | | **Nutrient:**  **Time** | |
| --- | --- | --- | --- | --- | --- | --- | --- | --- | --- | --- | --- | --- |
|  | F_1,8_ | P | F_1,8_ | P | F_6,48_ | P | F_1,8_ | P | F_6,48_ | P | F_6,48_ | P |
| Shannon  (zoo) | 1.036 | 0.338 | 0.894 | 0.372 | 25.36 | **<.001** | 5.082 | **0.028** | 1.481 | 0.205 | 1.405 | 0.232 |
| **Richness (zoo)** | 6.921 | **0.03** | 0.052 | 0.825 | 63.333 | <.001 | 9.520 | **0.015** | 3.733 | **0.004** | 1.184 | 0.330 |
| **Evenness (zoo)** | 6.045 | **0.02** | 6.340 | **0.01** | 1.923 | 0.09 | 10.096 | **0.002** | 0.280 | 0.943 | 0.544 | 0.772 |
| Biomass  (zoo) | 26.58 | **<.001** | 6.355 | **0.035** | 10.60 | **<.001** | 10.19 | **0.012** | 11.62 | **<.001** | 0.941 | 0.167 |
| Shannon  (phyto) | 1.474 | 0.259 | 16.37 | **0.003** | 37.45 | **<.001** | 0.051 | 0.827 | 2.504 | **0.034** | 3.748 | **0.003** |
| **Richness (phyto)** | 63.76 | **<.001** | 0.31 | 0.59 | 4.605 | <.001 | 12.45 | 0.007 | 4.268 | **0.001** | 1.051 | 0.404 |
| **Evenness (phyto)** | 6.792 | **0.03** | 15.914 | **0.004** | 16.887 | **<.001** | 1.227 | 0.3 | 4.051 | **0.002** | 2.906 | **0.01** |
| Biomass (phyto) | 31.59 | **<.001** | 25.55 | **<.001** | 131 | **<.001** | 7.75 | **0.023** | 0.134 | 0.724 | 20.931 | **0.002** |

*Shannon = Shannon Wiener diversity

**Table S2** One way ANOVA with repeated measures. Testing the effects nutrient addition and time on Shannon diversity, richnness, evenness and biomass of the **zooplankton (zoo)** and **phytoplankton** **(phyto)** communities at the **regional scale**. Significant p - values are in bold

| **Zoo** | **Nutrient** | | **Time** | | **Nutrient:**  **Time** | |
| --- | --- | --- | --- | --- | --- | --- |
|  | F_1,4_ | P | F_6,24_ | P | F_6,24_ | P |
| Shannon  (zoo) | 0.638 | 0.443 | 13.15 | **<.001** | 1.375 | 0.239 |
| **Richness (zoo)** | 3.226 | 0.14 | 19.37 | **<.001** | 1.394 | 0.257 |
| **Evenness (zoo)** | 0.544 | 0.502 | 1.345 | 0.276 | 1.277 | 0.305 |
| Biomass  (zoo) | 1.195 | 0.03 | 6.325 | **<.001** | 0.737 | 0.599 |
| Shannon  (phyto) | 16.05 | **0.002** | 27.23 | **<.001** | 3.053 | **0.011** |
| **Richness (phyto)** | 1 | 0.374 | 1 | 0.448 | 1 | 0.448 |
| **Evenness (phyto)** | 4.388 | 0.104 | 17.45 | **<.001** | 2.075 | 0.094 |
| Biomass  (phyto) | 3.344 | 0.141 | 56.45 | **0.002** | 8.068 | **0.047** |

*Shannon = Shannon Wiener diversity





**Fig. S1** Evenness and richness of phytoplankton at local scale (a) and (b); and at regional scale (c) and (d). In the graphs, metacommunities are represented by open diamonds and isolated communities are represented by filled circles. Continuous nutrient addition is represented by solid lines and pulsed nutrient addition is represented by dotted lines. Values are mean ± SE, n = 3





**Fig. S2** Evenness and richness of zooplankton at local scale (a) and (b); and at regional scale (c) and (d). In the graphs, metacommunities are represented by open diamonds and isolated communities are represented by filled circles. Continuous nutrient addition is represented by solid lines and pulsed nutrient addition is represented by dotted lines. Values are mean ± SE, n = 3

**
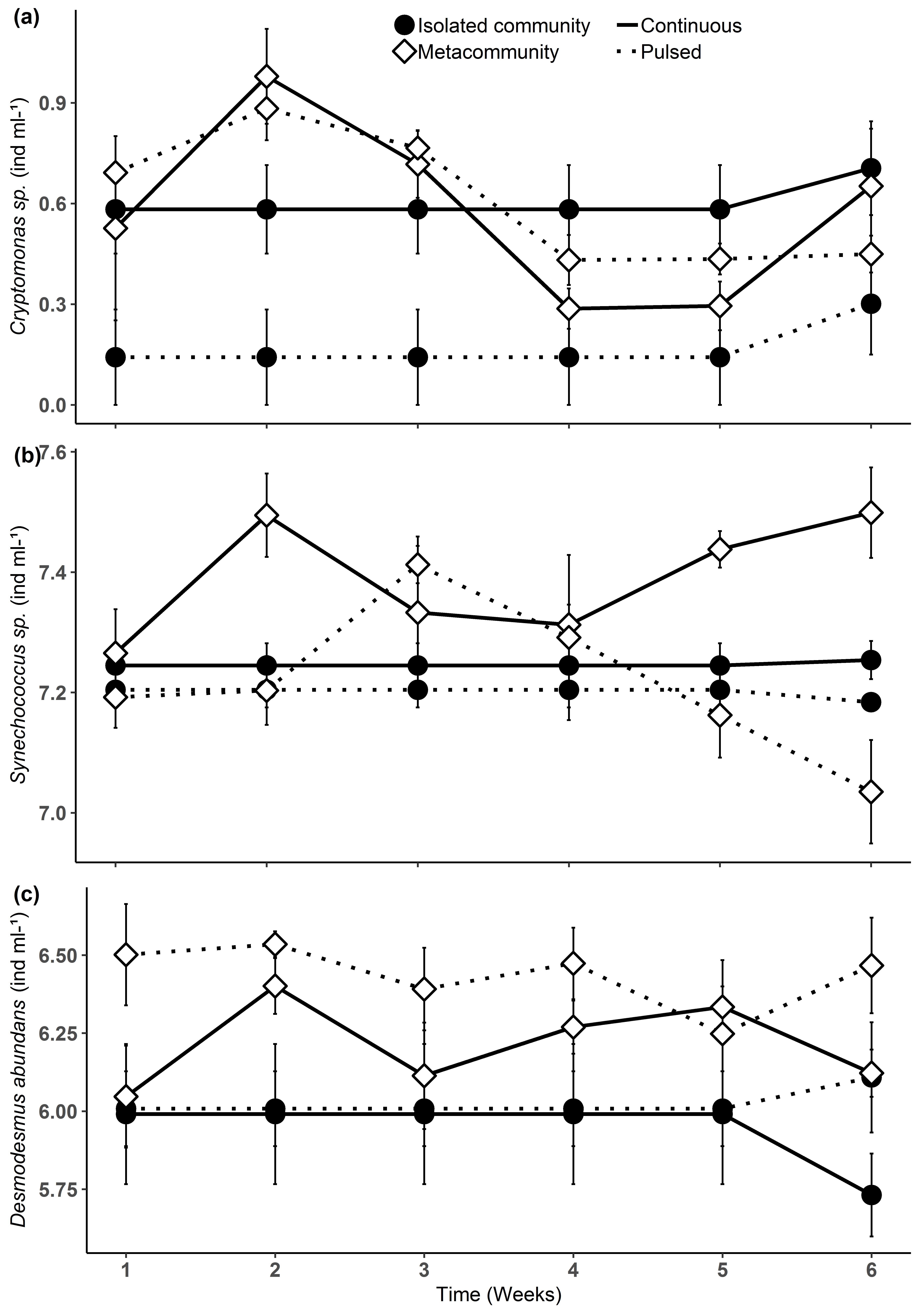
**

**Fig. S3** Abundance of each phytoplankton species over the experiment. Metacommunities are represented by open diamonds and isolated communities are represented by filled circles. Continuous nutrient addition is represented by solid lines and pulsed nutrient addition is represented by dotted lines. Values are mean ± SE, n = 3.Note log scale used in panel (log_10_ + 1)


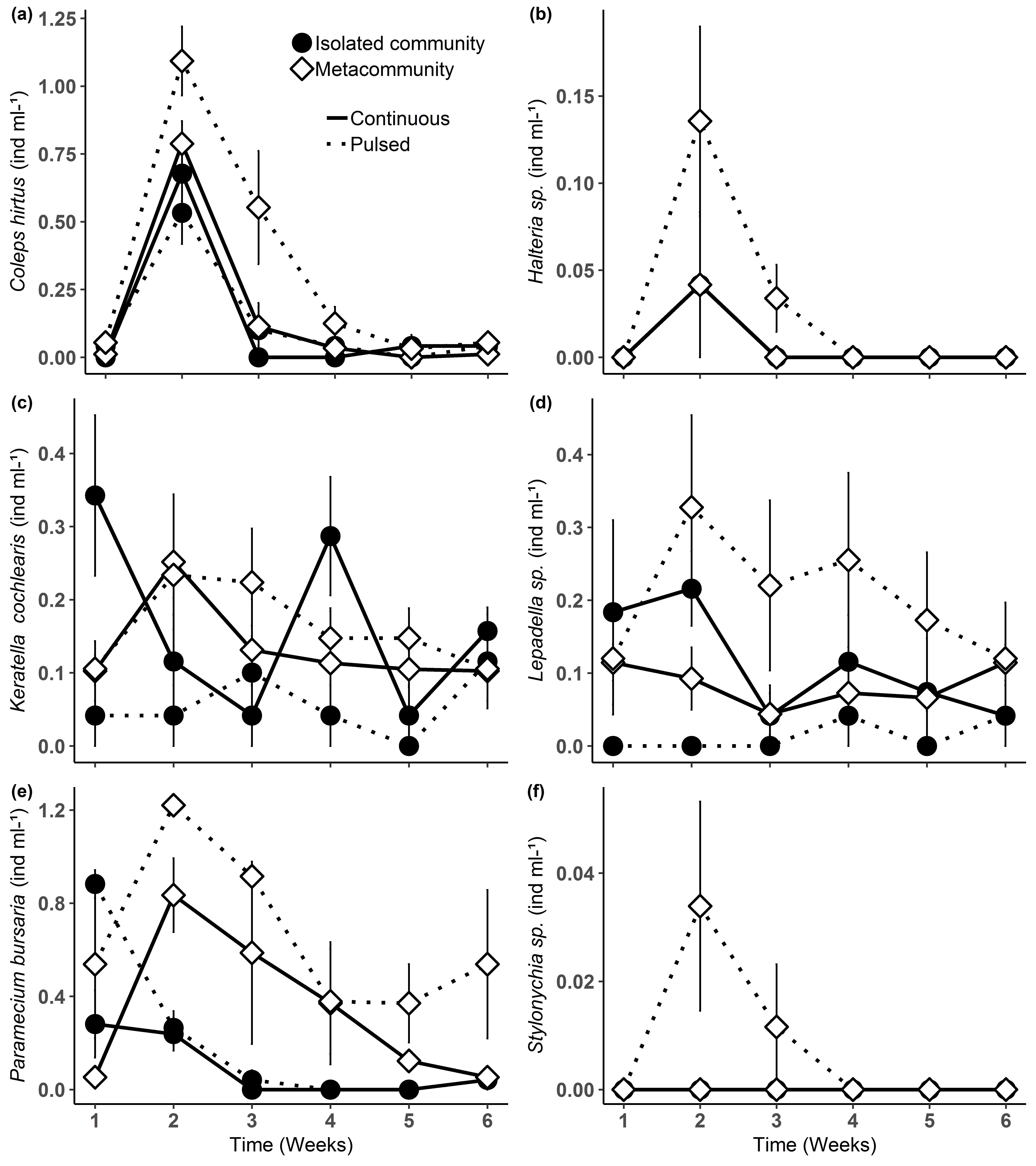


**Fig. S4** Abundance of each zooplankton species over the experiment. Metacommunities are represented by open diamonds and isolated communities are represented by filled circles. Continuous nutrient addition is represented by solid lines and pulsed nutrient addition is represented by dotted lines. Values are mean ± SE, n = 3.Note log scale used in panel (log_10_ + 1)


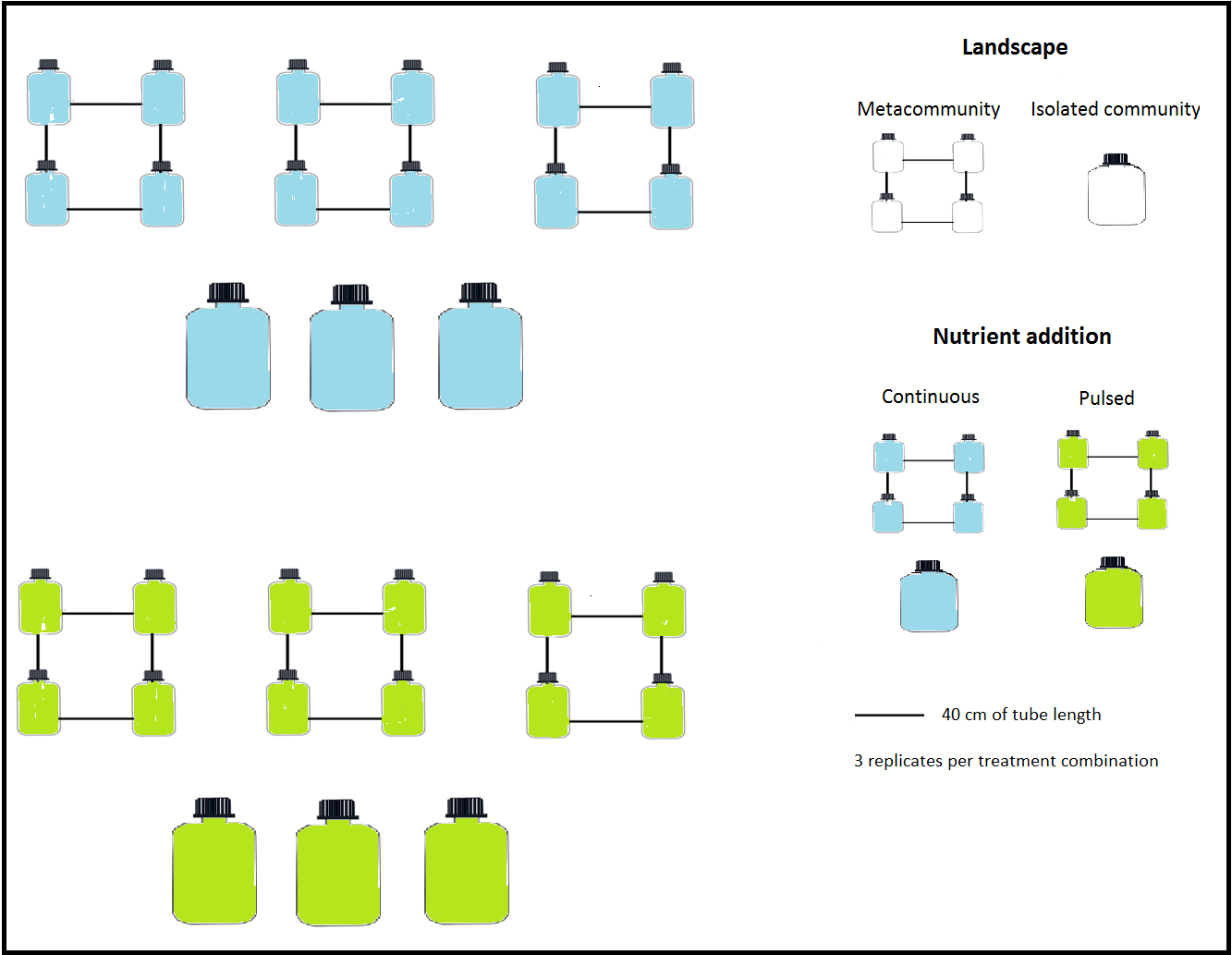


**Fig. S5** Experiment design (drawn picture)
